# A Case of Hepatotoxicity Induced by Therapeutic Ketamine Use for Sedation

**DOI:** 10.1155/2024/8366034

**Published:** 2024-03-12

**Authors:** Noah Yoo, Sarun Thomas, Michael Bender, Xian Jie Cindy Cheng

**Affiliations:** ^1^NYU Langone Health Long Island, Mineola, NY, USA; ^2^Northwell Health, New Hyde Park, NY, USA

## Abstract

Ketamine, initially developed as an anesthetic, has shown versatility in medical applications, including pain management, treatment-resistant depression, and sedation in the intensive care unit (ICU). While generally well-tolerated, long-term use at high doses raises concerns about potential toxicities, particularly in the liver. We present a case of a 27-year-old female with a complex medical history who received ketamine infusion for ICU sedation and experienced a sudden rise in liver function tests (LFTs), indicating possible ketamine-induced liver injury (KILI). The patient's liver function normalized after ketamine discontinuation. KILI is infrequent with short-term ketamine use, but emerging case reports suggest it may be associated with chronic or intermittent exposure. The underlying mechanisms for KILI are not fully understood but may involve the accumulation of ketamine metabolites, causing direct toxic effects on the liver. As ketamine's use expands, especially in critical care settings, clinicians should be vigilant for the potential development of KILI. Further research is needed to better understand its risk factors and mechanisms, as early detection and management of KILI are crucial to ensuring patient safety and optimizing ketamine's therapeutic benefits.

## 1. Introduction

Ketamine is an arylcyclohexylamine compound and N-methyl-D-aspartate (NMDA) receptor antagonist initially developed in the 1960s as an anesthetic that rapidly produces profound analgesia with a unique state of altered consciousness of limited duration effects of which could be extended with repeated administration [1]. Over the past several decades, ketamine was discovered to have potential beyond its anesthetic use, demonstrating value in diverse settings, such as pain medicine, treatment-resistant depression, and sedation in the intensive care unit (ICU). During the early stages of the SARS-CoV-2 (COVID-19) pandemic, ketamine was widely employed as an alternative sedative agent to allow for lung protective ventilation while patients received mechanical ventilation when other preferred agents were in short supply [2]. Although therapeutic use of ketamine is generally well tolerated and therefore widely used due to its limited safety profile compared to other sedatives [3, 4], long-term use at high doses may warrant consideration of its toxicities [5]. While it is not well known that ketamine may be associated with liver toxicities, it has been reported that prolonged use, repeated use, or abuse of ketamine may lead to liver injury [6, 7]. In light of its now more prevalent use, we report a case of potential ketamine-induced hepatitis in a patient who had exposure to long-term ketamine infusion for sedation.

## 2. Case Description

A 27-year-old female with a past medical history significant for trisomy 21 and autoimmune encephalomyelitis presented to the emergency department with a 4-day history of shortness of breath associated with fever and leukocytosis and was admitted with sepsis along with acute hypoxic respiratory failure. On hospital day 4, she deteriorated with worsening hypoxia, tachycardia, and acute encephalopathy requiring intubation and sedation. She was transferred to the ICU for the management of septic shock and acute respiratory distress syndrome (ARDS). The patient was treated with propofol and fentanyl to achieve deep sedation prior to the initiation of cisatracurium to facilitate the management of ARDS. On hospital day 8, her sedation was revised from propofol to ketamine due to concerns of persistent hypotension related to propofol.

The baseline aspartate aminotransferase (AST (5-34 IU/L)), alanine aminotransferase (ALT (0-37 IU/L)), and alkaline phosphatase (ALP (40-150 IU/L)) were 28, 11, and 88 IU/L, respectively. Over the ensuing 8 days, she required increasing amounts of ketamine to maintain sedation and ventilator synchrony. Eventually, she was able to transition off ketamine to dexmedetomidine and fentanyl. However, due to concerns of possible drug-related fevers caused by dexmedetomidine, ketamine was restarted 3 days later. On the morning of the 5th day of ketamine reintroduction, laboratory analysis showed an increase in her AST 160 IU/L, ALT 114 IU/L, and ALK 166 IU/L, while her total bilirubin remained normal. At this point, ketamine was administered at 2 mg/kg/hour or 94 mg/hour (Table 1). The daily dose of ketamine was 2256 mg on the day prior to identifying the elevation in the transaminase levels and 4138 mg over the total interval of ketamine reintroduction. LFTs were redrawn on the evening of the 5th day of ketamine reintroduction and showed worsening AST 211 IU/L, ALT 226 IU/L, and ALP 270 IU/L, while her total bilirubin remained normal.

**Figure 1:**
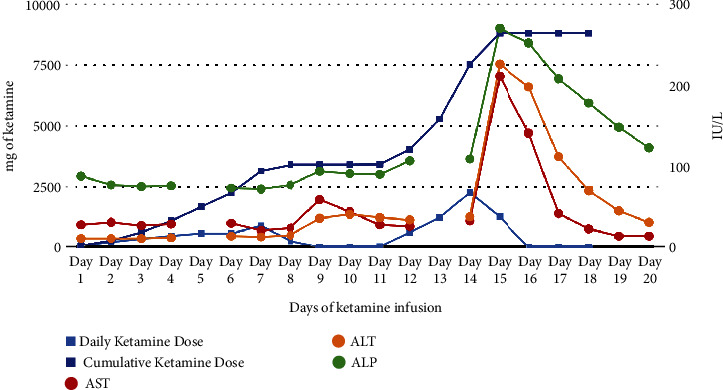
Trends in liver enzymes during ketamine infusion.

Drug-induced liver injury was considered the most likely explanation of her presentation, as prior LFTs were normal and her abdominal imaging up at that point showed normal liver morphology. Furthermore, at the time of her liver injury, the patient was well into her recovery from sepsic shock and had not required the administration of vasoactive medications to maintain her mean arterial blood pressure for many days. As ketamine was the only medication change during the 24 hours before the onset of hepatitis and was also rapidly titrated over this time frame, ketamine-induced liver injury (KILI) was suspected. Ketamine was discontinued over the next two days, and the patient was transitioned back to propofol and fentanyl. LFTs slowly trended down as the ketamine infusion was lowered and completely normalized 4 days after its discontinuation (Figure 1).

## 3. Discussion

Ketamine is extensively metabolized by the liver. Both isomers of ketamine are metabolized to norketamine by N-demethylation via CYP2B6, CYP3A4, and CYP2C9 [8]. Norketamine then undergoes further glucuronic acid conjugation and is eliminated by the kidneys [8]. The mechanism by which ketamine causes liver injury is not fully understood, but several hypotheses have been generated. The high concentrations of ketamine metabolites may cause direct toxic injury to surface epithelium and biliary epithelial cells with repeated use [7]. The factors that may contribute to this mechanism include mitochondrial dysfunction and increased lipid peroxidation, with the formation of free radicals, and allergic hepatitis [9]. Studies in rats that had prolonged ketamine infusion have demonstrated dose-dependent hepatotoxicity [10]. Prolonged ketamine infusions have been associated with mitochondrial degeneration in hepatic cells and dilations of the biliary tract and bile ducts [8]. Such findings are consistent with evidence from human case reports of dilation of the bile duct, histological evidence of bile duct injury, and liver fibrosis [6, 8]. In animal studies, ketamine has also been shown to induce hepatic CYP enzymes [11, 12], including CYP2E1 which may potentiate toxicities from other medications such as ethanol and acetaminophen by increasing the concentration of their toxic metabolites [13, 14]. The coingestion of ketamine with alcohol has been shown to increase the risk of hepatotoxicity [15].

In our case of a 27-year-old female with no past medical history of liver disease, ketamine toxicity was seen at a high cumulative dose (4.1 grams during the second infusion, 8.8 grams total) and high rate (96 mg/hour) of ketamine. The *R* factor for our patient was 3.4, showing mixed hepatocellular and cholestatic liver injury, consistent with other reports [6, 7, 9]. It is notable that toxicity occurred during the second infusion of ketamine, as other case reports have demonstrated LFT changes in reexposure of ketamine, but we believe that it may have more to do with prolonged and rapid exposure to ketamine during this second phase [6, 16]. Naranjo adverse reaction probability [17] was 8, a probable adverse drug reaction (Table 2). We determined that ketamine was the most likely cause of this adverse reaction not only due to the temporal relationship of rise and fall of LFTs as ketamine dose was weaned off but also because ketamine was the only medication that was increased in rate during the 24-hour period when LFTs abnormalities were noted. Other potential causes of hepatitis were considered but were eventually ruled out, including a comprehensive revision of the medication profile around the time of the injury (Supplement 1). The injury was not likely to be ischemic as the patient had been off of vasoactive medications for at least 24 hours. Sepsis was also unlikely given the patient's improvement from her infection. Viral origins were considered unlikely given the mild rise in LFTs. Other potential causes such as congestive hepatopathy and nonhepatic causes such as altered thyroid states and myopathy were also ruled out. In terms of medications, acetaminophen (1000 mg given 1 day prior to injury was deemed extremely unlikely to cause injury through theoretical interaction with ketamine), olanzapine (was on 5 mg twice daily), quetiapine (25 mg PRN; given 2 times on the day of injury), and propofol (was started the day prior to injury and was running at 20 mcg/kg/min) were considered unlikely as they did not have as strong temporal relationship with the hepatic injury. The exposure to acetaminophen, olanzapine, and quetiapine was low, and it did not elicit any response when it was given after the injury was resolved. Propofol has been shown in rare cases to cause hepatotoxicity as well [18–20], but given a relatively small dose (10-20 mcg/kg/min) and the fact that propofol was increased without further damage to LFTs allowed us to rule out propofol-induced liver injury. We have thoroughly assessed all the medications that were administered to the patient around the time of liver injury and have ruled out all potential other mechanisms, including potential drug interactions through ketamine's induction of CYP enzymes.

Ketamine is increasingly used for sedation in the ICU, and some patients may require prolonged sedation to manage their underlying disorder. KILI is considered rare during brief anesthesia. However, unusual biliary and hepatic complications have been described during chronic or intermittent use [7]. Wong et al. reported that among ketamine abusers (median weekly ketamine use 17.9 g ± 16), up to 9.8% had a liver injury [6]. Among those who had a liver injury, a biopsy revealed common bile duct dilation, microscopic bile duct injury, and significant liver fibrosis [6]. Noppers et al. reported 2 cases of liver injury in patients receiving S-ketamine infusion for complex regional pain syndrome (CRPS) [16]. The patients in that series received 2 separate ketamine infusions 16 days apart and the doses ranged from 1.5 g to 3.3 g during the first infusion and 1.4 g to 1.3 g during the second infusion. AST and ALT were elevated to 380 IU/L and 420 IU/L in one patient and 593 IU/L and 590 IU/L in the other patient, while it was reported that liver enzymes normalized after the discontinuation of ketamine infusion. Zhu et al. also published 2 cases of elevated liver enzymes in patients receiving ketamine infusion to treat pain from CRPS [9]. However, in this series, more than 7 months elapsed between ketamine doses in both patients. After infusions of 1254 mg and 1858 mg of ketamine, respectively, the liver enzymes sharply rose to ALT 1160 IU/L, AST 883 IU/L, and ALP 294 IU/L in one patient and ALT 746 IU/L, ALT 401 IU/L, and ALP 165 IU/L in the other patient. The second patient's presentation may have been confounded by chronic alcohol use. Both patients' liver enzymes trended down after discontinuation of the infusion. The Keta-Cov research group reported 5 patients who experienced elevated liver enzymes and cholangiopathy after exposure to intravenous ketamine during the COVID-19 pandemic [21]. The authors showed a linear relationship between the total intravenous dose of ketamine received and the maximum serum bilirubin level [21].

Although prior case reports have shown similar patterns of hepatotoxicity as we encountered in our case, ketamine was used for other indications—complex regional pain syndrome [9, 16] and abuse [6]—which may be associated with differing exposure and frequency. The accumulated ketamine dose was higher (4.1 g), and a faster rate (2 mg/kg/hr) was administered to our patient compared to other published case reports (accumulated: 1-2 g, maximum rate: 7.2 mcg/kg/hr) [9, 16]. A patient requiring a rapid or high cumulative dose of ketamine should be considered for a risk of hepatic injury. Furthermore, our case was not associated with sedation in COVID-19 [21], which has been linked to vasculobiliary injuries [22]. One patient in that report also had moderate alcohol consumption that may have confounded the liver injury [9]. We have thoroughly considered other causes, potential confounders, and comedications that could have contributed to this phenomenon in a patient without a prior history of liver dysfunction. A clear temporal relationship between the rate of ketamine infusion, total cumulative ketamine dose, and the timing of liver injury is evident in our case. Although the degree of exposure needed to show this injury may be different from individual patients, our report does indicate an increased need for monitoring when doses are titrated up rapidly or when patients require prolonged sedation. A prompt reduction in infusion rate and discontinuation of ketamine was commenced in our patient. It is not clear, however, if the injury would have further propagated or if such further injury were reversible had the infusion been continued, but due to the potential fibrotic nature of the injury and its likely pathophysiological mechanisms [6, 23], we decided that further use of ketamine should be avoided in this patient, given other options were present.

## 4. Conclusion

This case report underscores the importance of recognizing KILI as a potential complication in patients undergoing prolonged and high-dose ketamine infusion for sedation. The observed mixed hepatocellular and cholestatic liver injury aligns with previous reports, warranting vigilance in its clinical use. Although KILI is rare with short-term ketamine use, healthcare providers must be aware of this potential adverse effect and consider regular monitoring of liver function in patients on extended ketamine therapy. We draw attention and underscore the need for vigilance regarding another potential adverse effect of ketamine when used in high doses and for prolonged periods of sedation in critically ill patients.

## Figures and Tables

**Table 1:** Trends of ketamine dose and laboratory values.

	Daily ketamine dose (mg)	Cumulative ketamine dose (mg)	Ketamine rate (max mg/hr)	AST (IU/L)	ALT (IU/L)	ALP (IU/L)	Bilirubin, total (mg/dL)
Day 1	64	64	5	28	11	88	0.4
Day 2	210	274	9	31	11	77	0.4
Day 3	346	620	19	27	11	75	0.3
Day 4	473	1093	24	29	12	76	0.2
Day 5	576	1669	24	—	—	—	—
Day 6	576	2245	24	30	14	73	0.3
Day 7	896	3141	47	21	13	72	0.3
Day 8	254	3396	24	24	15	77	0.3
Day 9	0	3396	0	59	36	94	0.4
Day 10	0	3396	0	44	41	91	0.3
Day 11	22	3417	24	28	37	90	0.4
Day 12	620	4037	47	26	34	107	0.4
Day 13	1240	5277	94	—	—	—	—
Day 14	2256	7533	94	33	38	109	0.4
Day 15 (AM)	1270	8803	94	160	114	166	0.5
Day 15 (PM)				211	226	270	0.5
Day 16	0	8803	12	141	198	252	0.5
Day 17	0	8803	0	42	112	208	0.5
Day 18	0	8803	0	23	70	178	0.5
Day 19	0	8803	0	14	45	148	0.6
Day 20	0	8803	0	14	31	123	0.6

**Table 2:** Naranjo adverse drug reaction probability scale.

Question	Answer	Score
(1) Are there previous conclusive reports on this reaction?	Yes	+1
(2) Did adverse events appear after the suspected drug was given?	Yes	+2
(3) Did the adverse reaction improve when the drug was discontinued or a specific antagonist was given?	Yes	+1
(4) Did the adverse reaction appear when the drug was readministered?	Not known or not done	0
(5) Are there alternative causes that could have caused the reaction?	No	+2
(6) Did the reaction reappear when a placebo was given?	Not known or not done	0
(7) Was the drug detected in any body fluid in toxic concentrations?	Not known or not done	0
(8) Was the reaction more severe when the dose was increased, or less severe when the dose was decreased?	Yes	+1
(9) Did the patient have a similar reaction to the same or similar drugs in any previous exposure?	Not known or not done	0
(10) Was the adverse event confirmed by any objective evidence?	Yes	+1

Total: 8 probable ADR. The reaction followed a reasonable temporal sequence after a drug, followed a recognized response to the suspected drug, was confirmed by withdrawal but not by exposure to the drug, and could not be reasonably explained by the known characteristics of the patient's clinical state.

## Data Availability

The data that support the findings of this study are available on request from the corresponding author. The data are not publicly available due to privacy or ethical restrictions.

## Supplementary Materials

Supplementary Materials Supplement 1. Comprehensive medication profile around the time of liver injury.
