# Supplementary material for: A Case of Hepatotoxicity Induced by Therapeutic Ketamine Use for Sedation
Source: Case Rep Crit Care. 2024 Mar 12;2024:8366034. doi: 10.1155/2024/8366034 (PMC10950395; doi:10.1155/2024/8366034)
Supplement: Supplementary Materials — Supplement 1. Comprehensive medication profile around the time of liver injury. [file 8366034.f1.docx]

**Supplementary Material**

**Supplement 1. Comprehensive Medication Profile around the Time of Liver Injury**

| **Medications** | **Day**  **-6** | **Day**  **-5** | **Day**  **-4** | **Day**  **-3** | **Day**  **-2** | **Day**  **-1** | **Day**  **0** | **Day**  **+1** | **Day**  **+2** | **Day**  **+3** |
| --- | --- | --- | --- | --- | --- | --- | --- | --- | --- | --- |
| Acetaminophen PO | 1000 mg |  | 1000 mg | 1000 mg |  | 1000 mg |  |  |  |  |
| Dexamethasone IV |  |  | 10 mg |  |  |  |  |  |  |  |
| Dexmedetomidine IV | 1130.8 mcg | 1774.8 mcg | 1746.4 mcg | 199.6 mcg |  |  |  |  |  |  |
| Enoxaparin SQ | 140 mg | 140 mg | 140 mg | 140 mg | 140 mg | 140 mg | 140 mg | 140 mg | 140 mg | 140 mg |
| Etomidate IV | 20 mg |  |  |  |  |  |  |  |  |  |
| Famotidine IV | 40 mg | 40 mg | 40 mg | 40 mg | 40 mg | 40 mg | 40 mg | 40 mg | 40 mg | 40 mg |
| Fentanyl IV | 2000 mcg | 500 mcg | 1600 mcg | 2450 mcg | 2875 mcg | 3850 mcg | 4008.8 mcg | 4262.5 mcg | 5325 mcg | 4200 mcg |
| Fluoxetine PO | 60 mg | 60 mg | 60 mg | 60 mg | 60 mg | 60 mg | 60 mg | 60 mg | 60 mg | 60 mg |
| Furosemide IV | 40 mg | 40 mg | 40 mg |  |  |  | 20 mg | 60 mg | 80 mg | 120 mg |
| Haloperidol IV |  |  |  |  |  |  | 2 mg |  |  |  |
| Hydroxychloroquine PO | 400 mg | 400 mg | 400 mg | 400 mg | 400 mg | 400 mg | 400 mg | 400 mg | 400 mg | 400 mg |
| Levothyroxine PO | 112 mcg | 112 mcg |  | 112 mcg | 112 mcg | 112 mcg | 112 mcg | 112 mcg | 112 mcg | 112 mcg |
| Lorazepam IV |  |  |  |  |  |  | 1 mg | 1 mg | 1 mg |  |
| Magnesium sulfate IV |  |  |  |  |  |  | 2 g |  | 2 g |  |
| Melatonin PO | 10 mg |  | 10 mg | 10 mg | 10 mg | 10 mg | 10 mg | 10 mg | 10 mg | 10 mg |
| Meropenem IV | 3 g | 3 g | 3 g | 3 g | 3 g |  |  |  |  |  |
| Midazolam IV |  | 3 mg | 8 mg | 1 mg | 4 mg |  |  |  | 1 mg |  |
| Midodrine PO | 30 mg | 10 mg | 10 mg | 30 mg | 30 mg | 30 mg | 10 mg |  |  |  |
| Multivitamin PO |  |  |  |  | 15 mL | 15 mL | 15 mL | 15 mL | 15 mL | 15 mL |
| Norepinephrine IV | 4824.3 mcg | 6297.6 mcg | 5213 mcg | 7126.4 mcg | 8785.6mcg | 598.4 mcg |  |  |  |  |
| Olanzapine IM |  | 7 mg | 5 mg |  |  | 2 mg |  |  |  |  |
| Olanzapine PO |  |  |  |  |  | 10 mg | 10 mg | 10 mg | 10 mg | 10 mg |
| Piperacillin-tazobactam IV |  |  |  |  |  |  |  |  |  | 10.125 g |
| Potassium chloride PO | 40 mEq |  |  |  | 40 mEq |  | 40 mEq |  | 160 mEq |  |
| Ketamine IV |  |  | 22 mg | 620 mg | 1240 mg | 2256 mg | 1270 mg |  |  |  |
| Potassium phosphate IV |  |  |  |  |  | 15 mmol |  |  |  |  |
| Propofol IV |  |  |  |  |  | 226 mg | 900 mg | 540.8 mg |  | 282.8 mg |
| Quetiapine PO | 100 mg | 50 mg | 50 mg | 100 mg | 150 mg | 75 mg | 50 mg | 75 mg | 100 mg | 50 mg |
| Succinylcholine IV |  |  | 90 mg |  |  |  |  |  |  |  |
| Vancomycin PO | 500 mg | 250 mg | 125 mg | 250 mg | 250 mg | 250 mg | 250 mg | 250 mg | 250 mg | 250 mg |

Day 0 - day of liver injury

PO = per os; IV = intravenous; IM = intramuscular
